# Network Pharmacology and Experimental Verifications to Discover *Scutellaria baicalensis* Georgi’s Effects on Joint Inflammation, Destruction, and Pain in Osteoarthritis

**DOI:** 10.3390/ijms25042127

**Published:** 2024-02-09

**Authors:** Hee-Geun Jo, Chae-Yun Baek, Ho Sueb Song, Donghun Lee

**Affiliations:** 1 Department of Herbal Pharmacology, College of Korean Medicine, Gachon University, 1342 Seongnamdae-ro, Sujeong-gu, Seongnam-si 13120, Republic of Korea; jho3366@hanmail.net (H.-G.J.); cyning20@gachon.ac.kr (C.-Y.B.); 2 Naturalis Inc., 6 Daewangpangyo-ro, Bundang-gu, Seongnam-si 13549, Republic of Korea; 3 Department of Acupuncture & Moxibustion Medicine, College of Korean Medicine, Gachon University, 1342 Seongnamdae-ro, Sujeong-gu, Seongnam-si 13120, Republic of Korea

**Keywords:** *Scutellaria baicalensis* Georgi, network pharmacology, experimental verification, bioinformatics, osteoarthritis, East Asian herbal medicine

## Abstract

Osteoarthritis is the most common type of arthritis, characterized by joint pain and a decline in physiological function. *Scutellaria baicalensis* Georgi (SB) is potentially effective against osteoarthritis because of its wide range of anti-inflammatory pharmacological activities. This study aimed to identify the mode of action of SB against osteoarthritis using network pharmacology prediction and experimental verification. Networks were constructed to key compounds, hub targets, and pathways essential for SB’s effectiveness against osteoarthritis. Additionally, in vivo and in vitro tests were performed, including investigations on weight bearing in hind limbs, the acetic acid-induced writhing response, lipopolysaccharide-stimulated RAW264.7 cells, and serum cytokine responses. We identified 15 active compounds and 14 hub targets, supporting the anti-osteoarthritis effects of SB. The Kyoto Encyclopedia of Genes and Genomes pathway analysis indicated that fluid shear stress, atherosclerosis, phosphatidylinositol 3-kinase-Akt signaling, and cellular senescence pathways were important. SB showed substantial anti-inflammatory, analgesic, and joint tissue-protective effects against osteoarthritis. Our study shows that SB has the potential value to be further investigated as a candidate material for the treatment of osteoarthritis in the future.

## 1. Introduction

Osteoarthritis (OA) is the most common type of arthritis, characterized by joint pain and a decline in physiological function [1]. Recently, many countries have witnessed an increase in the socioeconomic costs associated with OA. According to a study utilizing Global Burden of Disease data, the annual age-standardized incidence rate of OA has increased by 0.32% globally [2]. In addition, more than 200 million people worldwide are already suffering from symptomatic and activity-limited OA [3]. In contrast, one-third of patients with OA have comorbidities due to causes, such as drug toxicity or inflammatory reactions, accompanied by an increased mortality rate (20%) [1]. The risk of OA, which substantially increases with age, will unavoidably become a serious medical issue in the near future, given that many nations are dealing with an aging population [4]. Previous studies have indicated that it is not difficult to forecast that as life expectancy increases, OA will become a larger societal burden as well as a considerable cause of financial distress and patient mortality. Although several interventions are currently available, additional research is required to develop highly effective treatments.

Although the course of OA varies greatly from patient to patient, most cases are characterized by symptoms such as pain, joint stiffness, reduced motor function, and muscle wasting [5]. Because it often has a long course, it is closely associated with worsening daily functioning, including psychiatric symptoms, sleep disturbances, and fatigue. Until recently, age-related degenerative changes in the musculoskeletal system that cause pain were thought to be the main pathophysiology of OA [5,6]. However, OA is not simply a local anatomical problem but a disease involving complex pathophysiology throughout the body [7]. Examples of therapeutic targets that can partially reverse the course of the illness, including persistent low-grade inflammation and chondrosenescence, have recently been highlighted in the pathogenesis of OA [8,9].

Currently, the recommended first-line treatment for OA is non-drug therapy, which includes physical activity, weight control, and patient education [1]. For drug therapy, topical nonsteroidal anti-inflammatory drugs are safe and effective [10]. If a patient does not respond to this treatment, further options include acetaminophen-containing oral medicine regimens, oral nonsteroidal anti-inflammatory drugs, opioid analgesics, serotonin-norepinephrine reuptake inhibitors, and intra-articular corticosteroid injections [1,4,11]. These medications have been used extensively, but there are concerns regarding a variety of side effects during long-term administration as well as the lack of efficacy in suppressing symptoms and altering the course of the disease [12]. In light of the existing evidence, there is a need to investigate new candidates that simultaneously target multiple pathogenic pathways and demonstrate a pharmacology that reduces disease progression in patients who present with a chronic pathology of OA that is not severe enough to require surgery. 

Natural products may emerge as new candidates for OA treatment due to their multi-compound/multitarget modes of action. According to a recent review of East Asian herbal medicines, multiple active compounds might simultaneously suppress OA pathologies, such as systemic inflammation, subchondral bone damage, cartilage destruction, synovial injury, and circulatory pathologic modification [13,14]. Based on these findings, a recently published population-based cohort study discovered that prescribing several East Asian herbal medicines to patients with OA may reduce the probability of joint replacement [15]. *Scutellaria baicalensis* Georgi (SB) has been used for the long-term treatment of inflammatory disorders in several East Asian countries, owing to its diverse pharmacological activities [16,17]. SB exhibits anti-inflammatory effects in addition to various other beneficial properties, including antioxidant, neuroprotective, immunomodulatory, hepatoprotective, anticancer, antibacterial, and antiviral effects [18,19,20,21]. Among these, the antioxidant and anti-inflammatory activities are considered the most important to effectively modulate low-grade inflammation-related multi-pathology, which is the main cause of progressive joint destruction in OA [22,23,24,25]. SB demonstrates potent anti-inflammatory activity and modulatory effects on autoimmune disease-mediated high-grade inflammation and inflammatory cytokines [26,27]. Its antioxidant activity and protective effects against various cardiovascular diseases have also been widely documented [24,28]. Therefore, it is reasonable to assume that SB is a promising agent for fundamentally inhibiting the long-term pathology of OA; however, the current literature on this topic is lacking. 

In the context of the above studies, we hypothesized that SB could be a useful candidate for inhibiting inflammation-induced pathology and pain progression in OA. Therefore, this study investigated whether SB is a promising multicomponent material that can ameliorate the multi-pathology of OA through computational prediction and experimental validation. For pharmacological prediction, we used network pharmacology analysis, a technique that predicts the complex relationships between multiple components and multiple targets in natural products from a network science perspective [29,30,31,32,33,34,35]. This method allows more precise predictive information to be derived about the drug–target–disease relationship, which provides the basis for designing optimized experiments to explore the mechanisms of action of multicomponent herbal medicines, including SB. In this study, we identified the main compounds in SB that are mainly responsible for mitigating OA and predicted the gene targets and signaling pathways through which they act. The predicted SB pharmacological network elements were cross-validated by inhibiting joint destruction and reducing pain in an in vivo animal model and by confirming anti-inflammatory effects in an in vitro cell model. Through these two steps, this study explored how the multicomponent anti-inflammatory pharmacology of SB could potentially contribute to alleviating OA symptoms and progression.

## 2. Results

### 2.1. Network Pharmacology of SB against OA

#### 2.1.1. Screening of Potential Active Compounds in SB for OA

**Figure 1:**
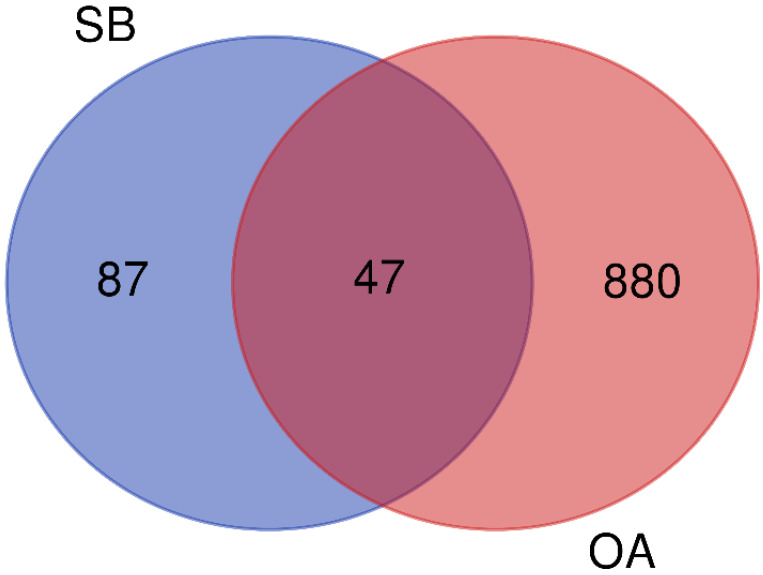
A Venn diagram showing potential targets for osteoarthritis treatment with *Scutellaria baicalensis*. SB: *Scutellaria baicalensis*, OA: osteoarthritis.

As a result of screening according to the criteria of OB ≥ 30% and DL ≥ 0.18 using four relevant databases, a total of 44 potential active compounds were found (Table 1). Approximately 87 compounds target association data from the DrugBank and the traditional Chinese medicine systems pharmacology database and the analysis platforms (TCMSP) were searched, while 880 human target genes associated with OA were acquired from the GeneCard database (relevance score ≥ 1). The 47 overlapping genes were identified as potential targets of SB against OA, when the targets of both SB and OA were ported into a Venn diagram (Figure 1). 

#### 2.1.2. Construction of D-C-T-D Network

**Figure 2:**
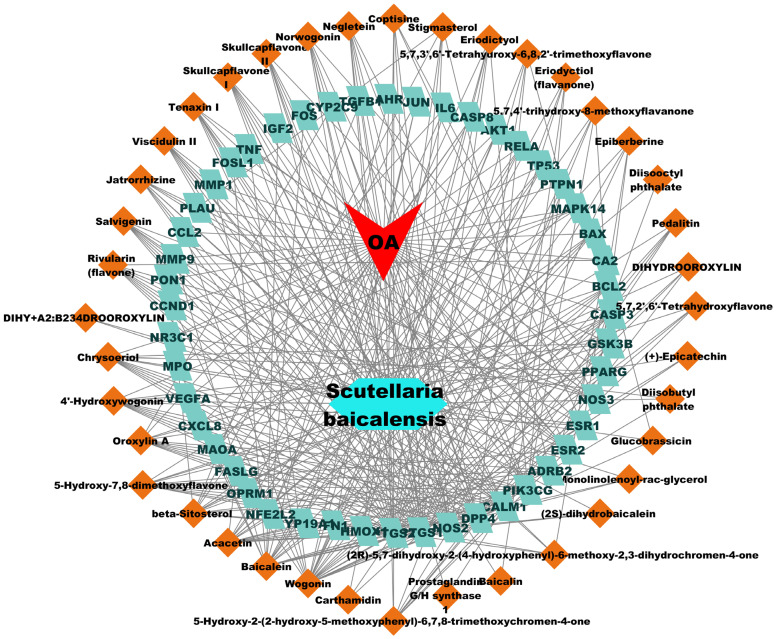
The therapeutic mechanisms of OA in SB are represented by the drug compound–target–disease network. OA: osteoarthritis, SB: *Scutellaria baicalensis*.

A total of 47 potential therapeutic targets from the common set of SB and OA targets were submitted to Cytoscape 3.9.1. Figure 2 illustrates the 78 nodes and 148 edges of the network. The number of linked nodes is represented by the degree of a single target in the D-C-T-D network. The network was assessed, and the levels of active compounds were scored using network analysis techniques. Compounds showing a high centrality exceeding 7.2564, the average of the degree of centrality, were as follows: wogonin (26), baicalein (19), acacetin (16), beta-sitosterol (13), 5-hydroxy-7,8-dimethoxyflavone (12), oroxylin a (12), 4′-hydroxywogonin (11), chrysoeriol (11), 5-hydroxy-2-(2-hydroxy-5-methoxyphenyl)-6,7,8-trimethoxychromen-4-one (10), rivularin (flavone) (9), jatrorrhizine (8), salvigenin (8), skullcapflavone I (8), tenaxin I (8), and viscidulin II (8) (Table 2).

#### 2.1.3. PPI Network Analysis

**Figure 3:**
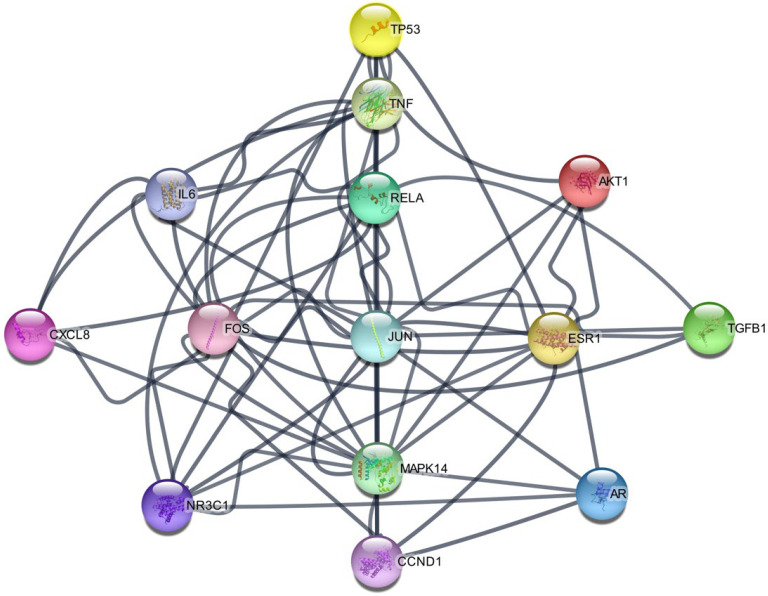
The PPI network of hub targets from SB against OA. PPI: protein–protein interaction; SB: *Scutellaria baicalensis*, OA: osteoarthritis.

The STRING 11.5 platform was used to import common targets, and a PPI network model (minimum required interaction score: 0.9) was generated by limiting the organism to “*Homo sapiens*”. The PPI network did not include four targets (ADRB2, CA2, MAOA, and OPRM1) because they did not interact with other targets. We identified 43 nodes with 129 edges and an average degree of 6.0. Nodes that satisfied the average degree centrality value (6.0) were retrieved through an additional examination of topological attributes, and 29 targets were eliminated during screening. Figure 3 shows the PPI network of the hub targets. Table 3 presents a list of hub targets with a high degree centrality of >6.0. 

#### 2.1.4. GO and KEGG Enrichment Analysis

**Figure 4:**
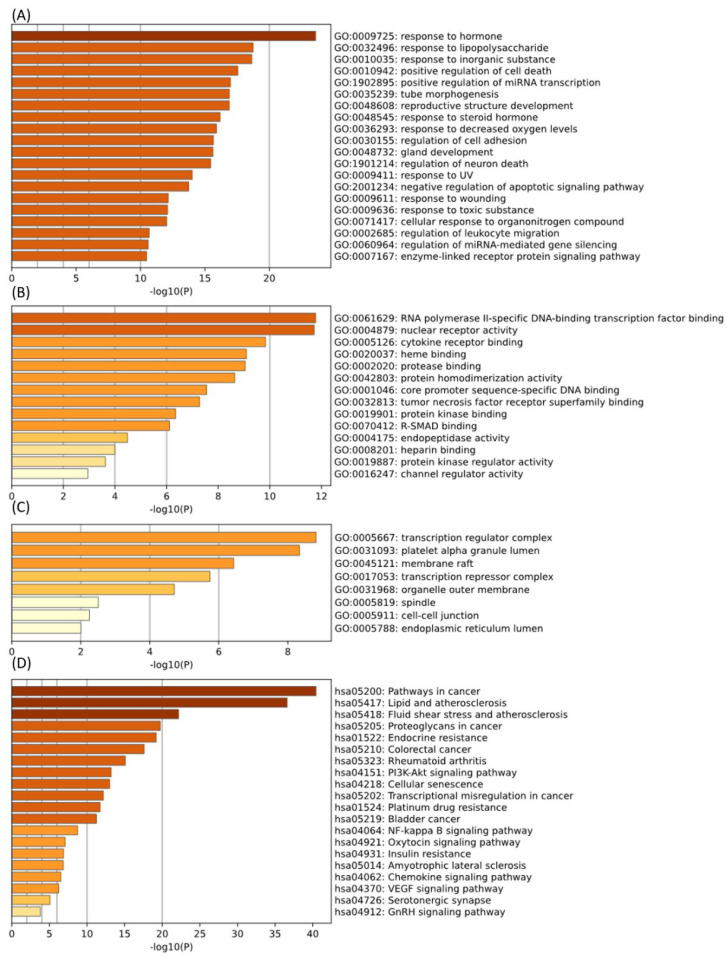
GO and KEGG enrichment analysis of OA treatment. ((**A**–**C**): GO analysis of OA targets. (**D**): KEGG enrichment analysis of OA targets). GO: Gene ontology, KEGG: Kyoto Encyclopedia of Genes and Genomes, OA: osteoarthritis.

The pharmacodynamic properties of the active compounds in SB were comprehensively represented using functional GO analysis. The Metascape platform was used to import common targets for the GO analysis. As a biological process, 901 items were identified, including the response to hormones, the response to LPSs, the response to inorganic substances, the positive regulation of cell death, the positive regulation of mRNA transcription, tube morphogenesis, reproductive structure development, the response to steroid hormones, the response to decreased oxygen levels, and the regulation of cell adhesion (Figure 4A). For molecular function, 75 items were identified, including RNA polymerase II-specific DNA-binding transcription factor binding, nuclear receptor activity, cytokine receptor binding, heme binding, protease binding, protein homodimerization activity, core promoter sequence-specific DNA binding, tumor necrosis factor receptor superfamily binding, protein kinase binding, and R-SMAD binding (Figure 4B). As a cellular component, 25 items were identified, including the transcription regulator complex, platelet alpha granule lumen, membrane raft, transcription repressor complex, organelle outer membrane, spindle, cell–cell junction, and endoplasmic reticulum lumen (Figure 4C).

A total of 143 pathways were identified in the KEGG pathway analysis. These findings demonstrate that most pharmacological mechanisms are associated with fluid shear stress and atherosclerosis. Additionally, endocrine resistance, rheumatoid arthritis, the PI3K-Akt signaling pathway, cellular senescence, the NF-κB signaling pathway, the chemokine signaling pathway, and the VEGF signaling pathway were important signaling centers of this mechanism (Figure 4D).

### 2.2. HPLC Analysis 

**Figure 5:**
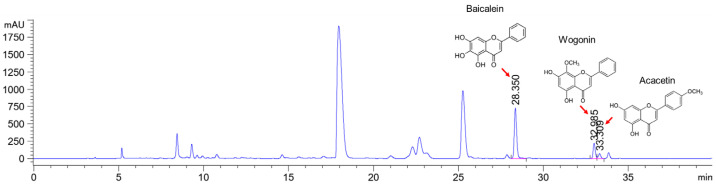
The high-performance liquid chromatogram of the *Scutellaria baicalensis* extracts at 275 nm: baicalein, wogonin, and acacetin. Retention times are 28.350 min, 32.985 min, and 33.309 min, respectively. The *x*-axis indicates the retention time; the *y*-axis indicates the absorbance unit. A Luna C18 column (250 mm × 4.6 mm, 5 μm; Phenomenex, USA) is used for chromatic separation at 30 °C.

Baicalein, wogonin, and acacetin were obtained from the SB and examined using HPLC-UV. The extract had a baicalein, wogonin, and acacetin content of 41.69 mg/g, 10.73 mg/g, and 9.71 mg/g, respectively. The HPLC chromatograms of the analysis following the chemical structures of the compounds are shown in Figure 5. The retention times of baicalein, wogonin, and acacetin were 28.35 min, 32.99 min, and 33.31 min, respectively.

### 2.3. Effects on the Weight-Bearing Arrangement in MIA Rat Models

**Figure 6:**
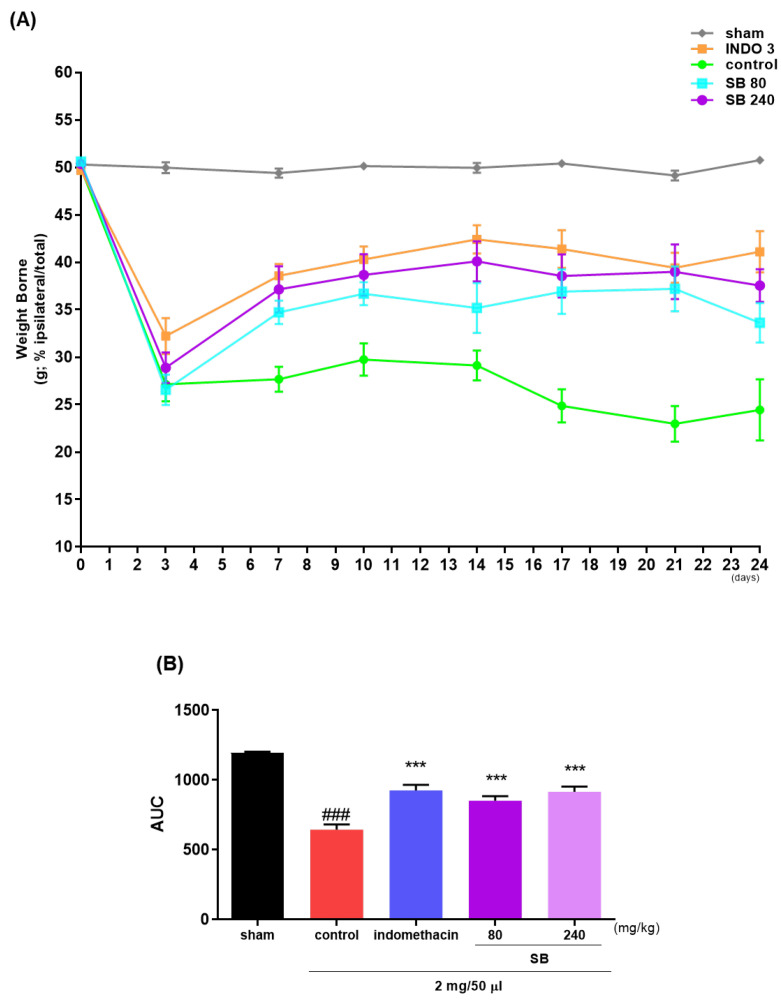
The effects of SB on hind limb weight bearing in an osteoarthritis rat model induced by monosodium iodoacetate. (**A**) The weight-bearing ratio of monosodium iodoacetate rats treated with 3 mg/kg indomethacin and 80 and 240 mg/kg SB during a period of 0–24 days was examined, and (**B**) the incapacitance meter tester examined the AUC. *** *p* < 0.001 compared. control, ### *p* < 0.001 vs. sham. INDO 3: indomethacin 3 mg/kg, SB: *Scutellaria baicalensis*, AUC: area under the curve.

The weight bearing of the hind limb is an index of joint inconvenience and pain and is used to estimate the analgesic effects of natural products on OA in rat models. The weight-bearing ratios of the left and right limbs were recorded for 24 days. The weight-bearing ratio of the control group significantly decreased on day 7 (Figure 6A) and remained lower thereafter, in contrast to that of the sham group. Simultaneously, the SB administration increased the weight-bearing capacity in the MIA group. In particular, the improvement in weight bearing with 240 mg/kg SB was similar to that observed with indomethacin (Figure 6B). 

### 2.4. Cartilage Damage in the MIA Model

**Figure 7:**
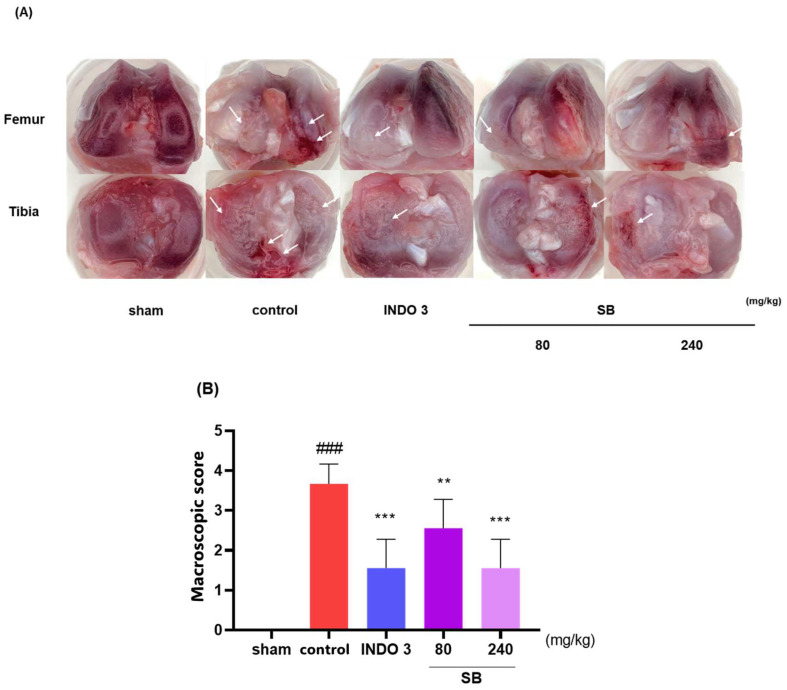
Photographs of the knee joint cartilage of rats with osteoarthritis induced by MIA. (**A**) The representative image indicates cartilage erosion with 3 mg/kg indomethacin, and 80 and 240 mg/kg SB were administered to MIA rats. The arrows indicate the cartilage degradation spot. (**B**) The macroscopic score. ### *p* < 0.001 vs. sham, ** *p* < 0.05 vs. control, *** *p* < 0.001 vs. control by a one-way analysis of variance, Dunnett’s test. INDO 3: indomethacin 3 mg/kg, MIA: monosodium-iodoacetate, SB: *Scutellaria baicalensis*.

Photographs of the knee joint cartilage in each group show that SB suppressed cartilage damage induced by MIA injection. As shown in Figure 7A, the cartilage of the sham rats was glossy and lustrous, whereas the control rat cartilage was less shiny and rougher, with damage in some parts. Cartilage damage was significantly improved in SB- and indomethacin-treated rats following the macroscopic score (Figure 7B). Notably, recovery from cartilage damage by SB was similar to that by indomethacin.

### 2.5. Inflammatory Cytokine Levels in MIA Rats

**Figure 8:**
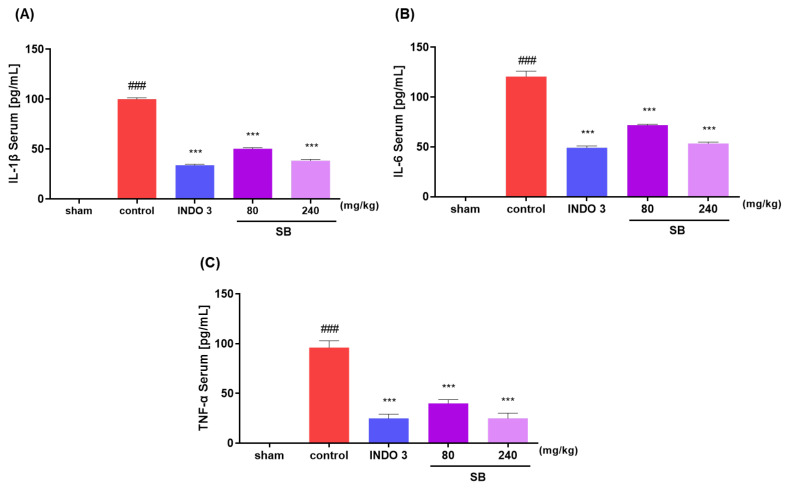
(**A**) The IL-1β, (**B**) IL-6, and (**C**)TNF-α serum of MIA rats. Rats were treated with 80 and 240 mg/kg SB for 24 d. ### *p* < 0.001 vs. sham, *** *p* < 0.001 vs. control by a one-way analysis of variance, Dunnett’s test. INDO 3: indomethacin 3 mg/kg, MIA: monosodium-iodoacetate, SB: *Scutellaria baicalensis*.

The IL-1β, IL-6, and TNF-α levels in the rats were analyzed after separating the serum gathered from each group. The SB-administered group indicated a significant decrease in the concentrations of IL-1β, IL-6, and TNF-α in the serum in a dose-dependent manner compared to that of the control group. Notably, 240 mg/kg SB decreased inflammatory cytokine levels, similar to those observed in the indomethacin 3 mg/kg rats (Figure 8).

### 2.6. Effect on Analgesic Responses

**Figure 9:**
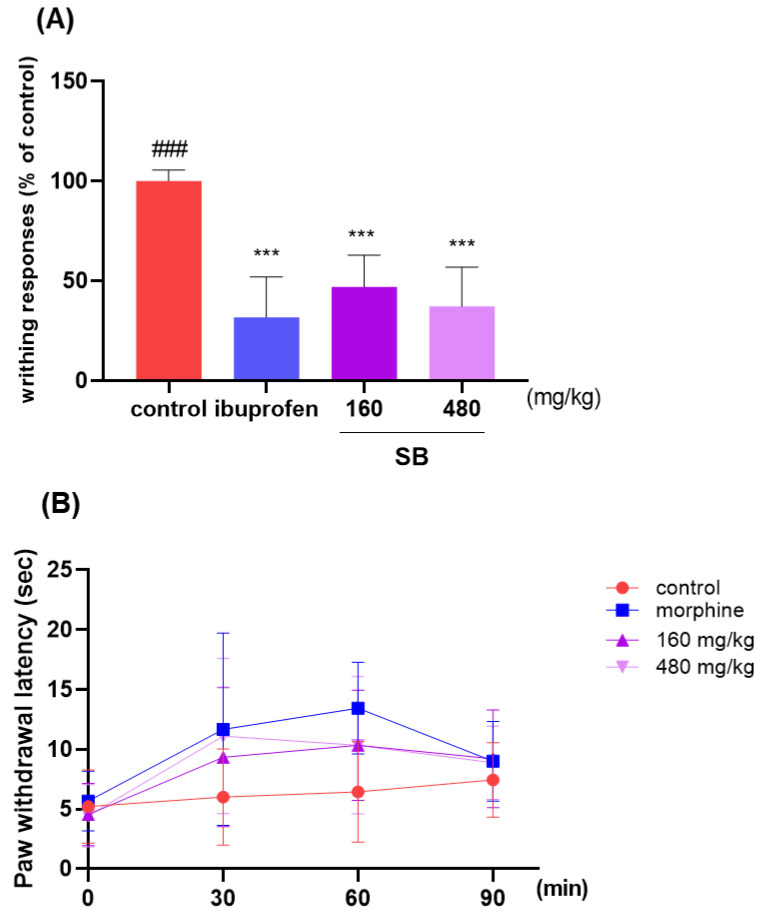
The effect on analgesic responses. (**A**) After 30 min of oral administration with ibuprofen (200 mg/kg body weight) and SB extract (160 and 480 mg/kg body weight), all mice were intraperitoneally injected with 0.7% acetic acid 10 min before counting. The number of writhing responses in acetic acid-induced ICR mice was eight per group; ### *p* < 0.001 vs. ibuprofen, *** *p* < 0.001 vs. control by a one-way analysis of variance, Dunnett’s test. (**B**) Hot plate responses of paw withdrawal latency. SB: *Scutellaria baicalensis*.

The analgesic effects of SB were investigated based on the writhing response of the acetic acid-induced mice and the hot plate test. The average writhing number in the control mice after 10 min was 100. SB administration resulted in a reduced number of writhing events compared to that in the control group. Mice fed with 480 mg/kg SB had an average writhing number of 37.15, which was lower than that of the positive control group (31.65). This result indicated the analgesic effects of SB (Figure 9A). The paw withdrawal latency was measured using a hot plate at 30, 60, and 90 min from 30 min after sample administration, morphine, SB 160 and 480 showed differences at 30 and 60 min and no difference at 90 min compared to control. In this study, morphine was analgesic at 60 min post-dose, while SB was most analgesic at 60 min post-dose at 160 mg/kg and at 30 min post-dose at 480 mg/kg. The paw withdrawal latency at 30 min at 480 mg/kg SB was 11.11 s, while the positive control morphine was 11.67 s, indicating similar analgesic effects (Figure 9B).

### 2.7. Anti-Inflammatory Effects in LPS-Stimulated RAW264.7 Cells

**Figure 10:**
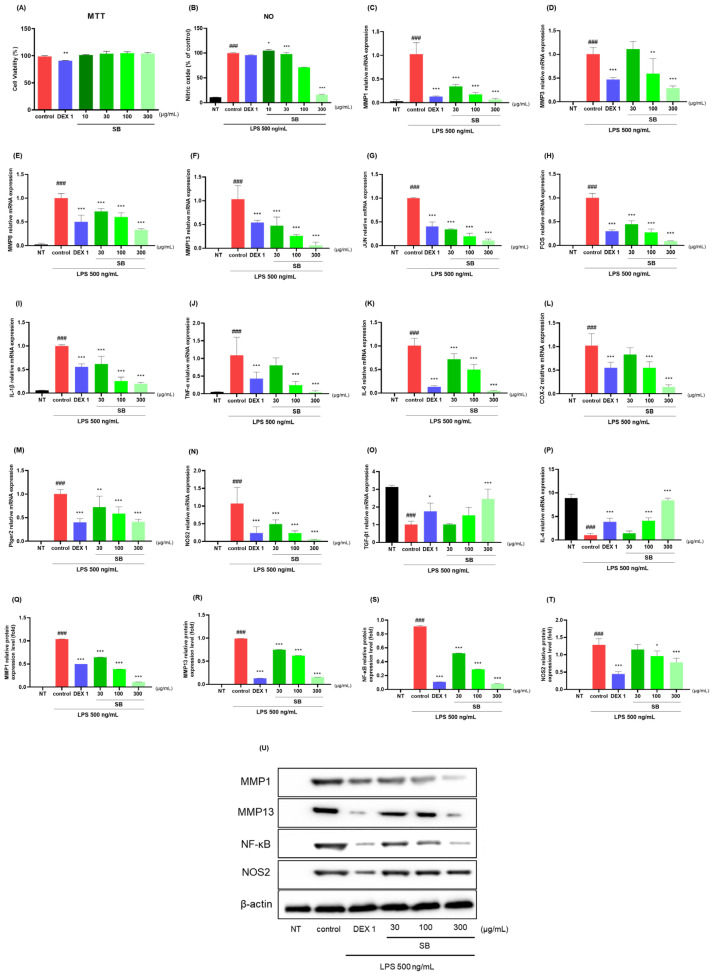
Effects of SB on (**A**) cell viability and (**B**) LPS-stimulated NO production, (**C**–**T**) mRNA (MMP-1, 3, 8, and 13), JUN, FOS, IL-1β, TNF-α, IL-6, COX-2, Ptger2, NOS2, TGF-β1, and IL-4, and the protein expression of (**U**) MMP-1, MMP-13, NF-κB p65, and NOS2 in RAW264.7 cells. Cells were treated with dexamethasone (1 µg/mL), SB (10, 30, 100, and 300 µg/mL), and LPS (500 ng/mL) for 24 h. ### *p* < 0.001 vs. sham, * *p* < 0.05 vs. control, ** *p* < 0.01 vs. control, *** *p* < 0.001 vs. control by a one-way ANOVA, Dunnett’s test. DEX 1: dexamethasone 1 µg/mL, LPS: lipopolysaccharide, NO: nitric oxide, NT: non-treated, ANOVA: analysis of variance.

The anti-inflammatory effects of SB were estimated in LPS-stimulated RAW264.7 cells using qRT-PCR and Western blotting. Up to 300 µg/mL, SB had no potential cytotoxic effects on RAW264.7 cells (Figure 10A). Decreased NO concentration, qRT-PCR, and protein expression levels were observed. SB reduced the LPS-stimulated NO production in a dose-dependent manner. Notably, 300 µg/mL SB was analyzed at 16.06, which achieved an 84% reduction in NO compared to that in the control group (Figure 10B). As shown in Figure 10C–P, SB and dexamethasone 1 µg/mL reduced the mRNA expression level of MMPs (MMP-1, 3, 8, and 13), JUN, FOS, IL-1β, TNF-α, IL-6, COX-2, Ptger2, NOS2, TGF-β1, and IL-4. Western blotting was used to analyze the anti-inflammatory effects of SB in LPS-induced RAW264.7 cells. As shown in Figure 10Q, the protein expression of the proinflammatory cytokines and mediators, such as MMP-1, MMP-13, NF-κB p65, and NOS2 were repressed by SB treatment in LPS-stimulated RAW264.7 cells. As illustrated by the Western blot images, SB reduced the expression of MMP-1, MMP-13, NF-κB p65, and NOS2 in a dose-dependent manner. Noticeably, 300 µg/mL SB indicated higher anti-inflammatory effects against all three cytokines than the positive control. 

### 2.8. Effects on Cytokine Responses in Knee Joint Cartilage Tissue

**Figure 11:**
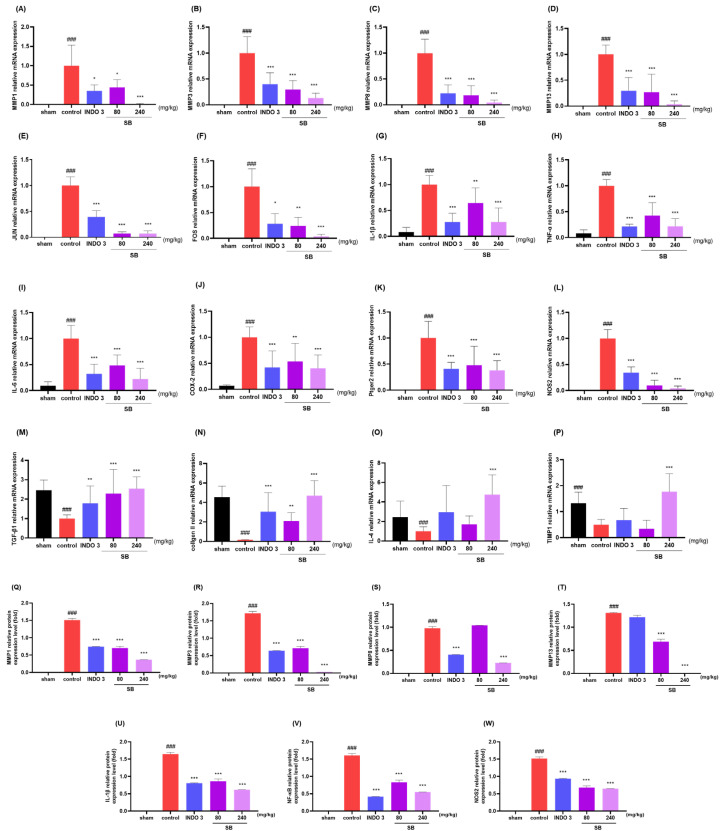

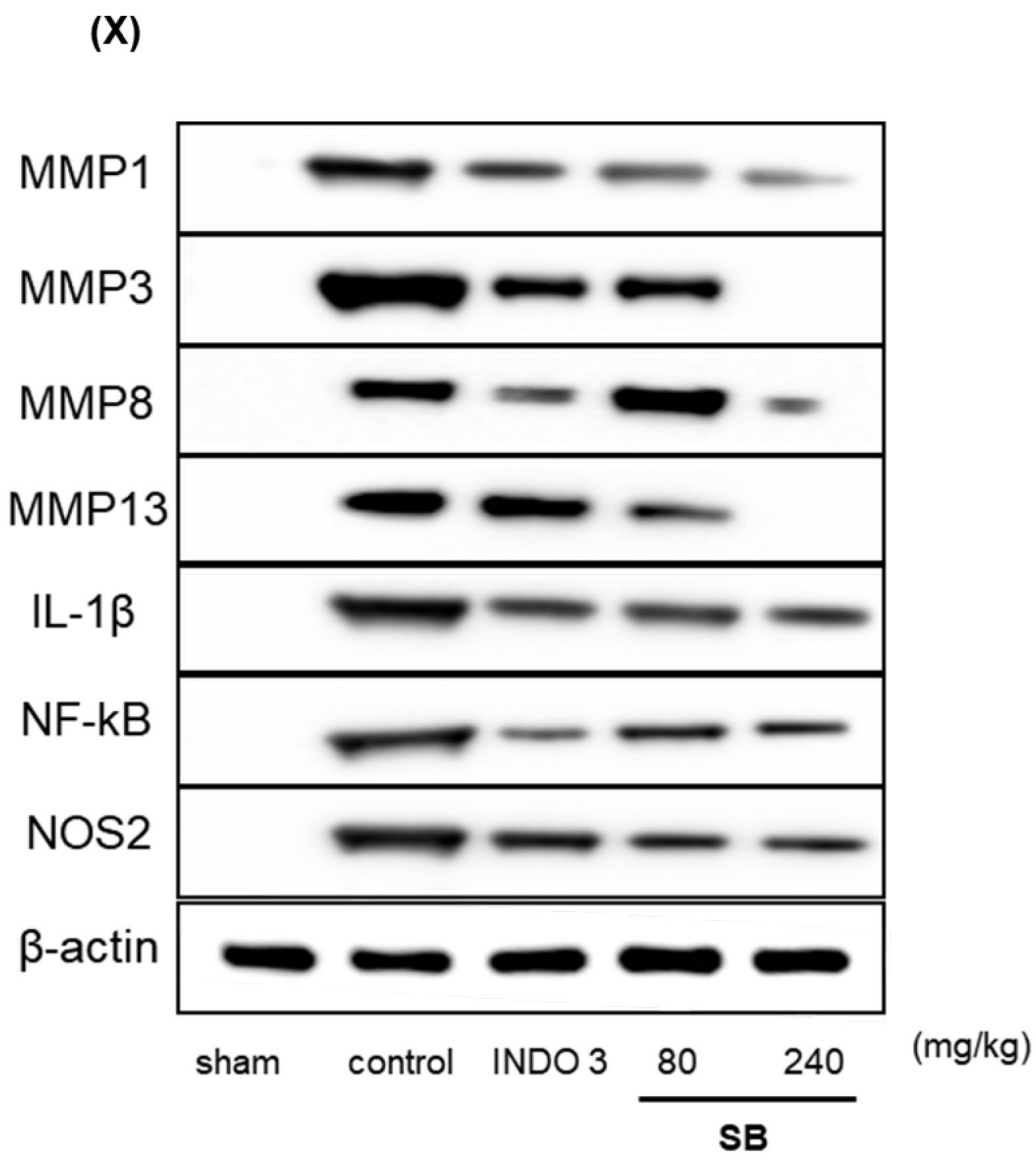
Changes of cytokines at knee joint cartilage tissue with 3 mg/kg indomethacin and 80 and 240 mg/kg SB treatment. (**A**–**W**) The mRNA expression of matric metalloproteinases (MMPs) (MMP-1, 3, 8, and 13), JUN, FOS, IL-1β, TNF-α, IL-6, COX-2, Ptger2, NOS2, TGF-β1, type II collagen, IL-4, and TIMP-1 determined by quantitative real-time PCR. (**X**) The protein expression of MMPs (MMP-1, 3, 8, and 13), NF-κB p65, NOS2, and IL-1β measured with Western blot analysis. ### *p* < 0.001 vs. sham, * *p* < 0.05 vs. control, ** *p* < 0.01 vs. control, *** *p* < 0.001 vs. control by a one-way ANOVA and Dunnett’s test. INDO 3: indomethacin 3 mg/kg, MIA: monosodium-iodoacetate, ANOVA: analysis of variance, SB: *Scutellaria baicalensis*, PCR: polymerase chain reaction.

The analysis of the mRNA levels of MMPs (MMP-1, 3, 8, and 13), JUN, FOS, IL-1β, TNF-α, IL-6, COX-2, Ptger2, TGF-β1, type II collagen, IL-4, and TIMP-1 in the rats indicated that SB administration significantly decreased the MMPs (MMP-1, 3, 8, and 13), JUN, FOS, IL-1β, TNF-α, IL-6, COX-2, Ptger2, and NOS2 levels (Figure 11A–L), while increasing TGF-β1, type II collagen, IL-4, and TIMP-1 (Figure 11M–P) in knee joint cartilage tissue compared to that in the control rats. Noticeably, 240 mg/kg SB rats had lower levels of MMP-1, MMP-3, JUN, FOS, IL-1β, and TNF-α and higher levels of type II collagen and IL-4 than in the indomethacin rats. The Western blot analysis also identified SB’s downregulating effects on MMPs (MMP-1, 3, 8, and 13), NF-κB, IL-1β, and NOS2 in MIA rats (Figure 11Q).

## 3. Discussion

In this study, we predicted that SB could exhibit anti-inflammatory and anti-nociceptive activities against OA based on network pharmacology analysis and experimental verification. SB increased the weight-bearing capacity and prevented cartilage damage in MIA rats. In addition, a significant pain-suppressive effect was confirmed by observing the writhing response of acetic acid-treated mice. Meanwhile, in LPS-induced RAW264.7 macrophages, inflammatory cytokines MMPs, IL-1β, TNF-α, IL-6, COX-2, Ptger2, JUN, and FOS were inhibited, and anti-inflammatory factors, TGF-β1, type II collagen, and IL-4, enhanced the activity of TIMP-1. To the best of our knowledge, this is the first study to analyze the bioactivity of SB as a single material against OA using an integrated research method. SB not only exhibits a wide range of pharmacological actions but also has excellent anti-inflammatory effects. Considering that persistent synovial inflammation in OA has recently been identified as an important therapeutic target, the findings of this study are expected to enhance the potential of SB as a promising candidate for mitigating OA.

Network Pharmacology identified 15 possible SB-active compounds, including wogonin, baicalein, acacetin, beta-sitosterol, oroxylin A, and chrysoeriol. HPLC analysis revealed that wogonin, baicalein, and acaetin were the main active ingredients. These results specifically support the effect of SB on OA in light of previous studies. Wogonin inhibited the production and expression of inflammatory mediators, including IL-6, COX-2, Ptger2, iNOS, and NO, in IL-1-stimulated OA chondrocytes via the ROS/ERK/Nrf2 pathway [36]. This suggests that wogonin exerts potent anti-inflammatory and chondroprotective effects against OA. In addition, the tetrahedral framework nucleic acid/wogonin complex not only increases the expression of chondrogenic markers but also inhibits apoptosis and inflammatory mediators and is a potential candidate material for OA injections [37]. Baicalein is expected to be a potential treatment for the progressive course of OA by suppressing chondrocyte ferroptosis and improving AMPK/Nrf2/HO-1 signaling activity [38]. This effect is also consistent with other studies showing that baicalein exerts a therapeutic effect on OA by suppressing the expression of apoptosis-related proteins through its action on the apoptotic signaling pathway [39]. In contrast, acacetin can exhibit therapeutic activity against OA by inhibiting NF-κB signaling pathways and suppressing the IL-1β-induced expression of MMPs in chondrocytes [40]. Furthermore, acacetin exhibited therapeutic effects on arthritis, an autoimmune disease, by preventing pathological alterations in a murine model of collagen-induced arthritis and suppressing Th17 cell generation [41]. Meanwhile, we predicted that the effect of SB on OA through network pharmacology would be mediated by 14 hub targets. The in vitro results of this study confirmed that SB treatment was associated with significantly reduced mRNA expression levels of the proinflammatory and inflammatory cytokines JUN, FOS, MMP-1, MMP-3, MMP-8, MMP-13, IL-6, IL-1β, COX-2, and TNF-α compared to those in the control group. In particular, the mRNA expression levels of JUN, MMP-1, MMP-3, IL-1β, and TNF-α were significantly reduced in the SB treatment group compared to that in the positive control, indomethacin. The herbal targets predicted in this study are closely related to OA and are considered to constitute a distinct subset of its pathology. Therefore, it is reasonable to expect a wide range of indications for SB in OA owing to the action of multiple compounds on multiple targets. The active compounds and hub targets of SB predicted and validated in this study are multiple, and they are all closely associated with improving OA pathology. Hence, we can conclude that the extensive effects of SB in OA-simulated in vitro and in vivo experiments were due to multicomponent/multitarget effects.

In the in vivo experiment, the MIA rat group administered SB daily for 24 days showed a significant increase in hindlimb weight-bearing capacity compared to that in the control group. In addition, after seven days of ingestion, the weight-bearing capacity was not inferior to that of indomethacin, the positive control group, and this effect was expressed in a dose-dependent manner. In addition, significant recovery of medial and lateral tibial cortical damage was observed in the SB-administered group compared to that in the control group. This indicates that SB can exhibit a wide range of therapeutic effects on pathological changes in the cartilage and subchondral bone in addition to simple analgesic and anti-inflammatory effects in OA. In the writhing response measurement results for evaluating peripheral pain relief efficacy, significant effects were observed in the SB-administered group at all doses compared to the control group in a dose-dependent manner. In addition, the serum and cartilage tissues of the MIA rats were separated to confirm the expression of cytokines and inflammatory mediators. As a result, the mRNA expression level of proinflammatory and inflammatory cytokines in the SB intake group was significantly decreased compared to that in the control group, and the anti-inflammatory factors, such as TGF-β1, Collagen II, IL-4, and TIMP-1 mRNAs, were increased. This mechanism was confirmed using an LPS-induced RAW264.7 cell inflammation model. SB significantly suppressed the LPS-induced overproduction of NO and inflammatory cytokines, such as IL-1β, IL-6, iNOS, and COX-2, in this experiment. The positive findings of the in vivo experiments in this study may be interpreted as indicating the bioactivity of SB, which is consistent with recent studies showing that reducing early OA inflammation can disrupt the vicious cycle of joint degeneration and destruction [18,42].

Our findings suggest the need for further follow-up studies in several aspects. Firstly, the anti-inflammatory and antioxidant activities of SB that inhibit the progressive pathology of OA should be examined in greater detail. The anti-inflammatory activity of SB is well established [18,26,27,43]. In contrast, the antioxidant activity of SB mediates protective effects in biological tissues, such as myocardial damage in myocardial ischemia and the inhibition of oxidative stress-induced liver injury; its antioxidant capacity is also stronger than that of other widely studied materials [24,28,44,45]. Therefore, the OA-related suppressive and chondroprotective effects of SB observed in this study may be attributed to both anti-inflammatory and antioxidant mechanisms. The challenge for further experimental studies is to explore the optimal conditions for the anti-inflammatory and antioxidant effects of SB to inhibit the long-term destructive joint pathology of OA. In particular, the design of novel formulations, such as nanocarriers, to improve the low bioavailability of natural products is important to extend the results of the present study [46,47]. Second, to specify the polypharmacology-based efficacy of natural products, such as SB, it is necessary to identify the pathways through which the greatest effect may ultimately be expressed. To this end, we used GO and KEGG enrichment analyses to predict different pathways. However, for experimental validation, the pathway was restricted to NF-κB. This selection was not based on the dominance of a particular pathway but rather on the reproducibility of the experimental design, as it is the most widely used pathway for inflammation and joint tissue protection in OA in natural product studies [48,49,50,51,52,53]. Therefore, based on the positive results of this study, further experiments on the dominance of multiple pathways as individual targets and the conditions under which synergistic enhancement of effects occurs are needed to further investigate the polypharmacological disease-suppressing effects of SB on OA. Information on the signaling pathways derived from network pharmacology predictions in this study is expected to be useful for further research.

Based on an integrative approach, the study initially predicted the mode of action of SB through network pharmacology analysis and then validated the predictions using in vivo and in vitro models of OA. However, this study has some limitations. First, the multifaceted effects of SB observed in the in vivo OA model have not been fully elucidated in terms of the compounds, targets, and pathways that have been specifically optimized based on their effects. To compensate for this, a multi-compound/multitarget/multi-pathway network was identified in advance by bioinformatics analysis; however, it was difficult to identify all of these in a single experimental study. Nevertheless, we believe that this study confirms the potential of SB as a disease-modifying drug candidate for OA, and we will continue to conduct follow-up experiments to validate additional mechanisms and ranges of action based on these results. Second, the pharmacological effects that ultimately inhibit the disease process of OA are derived from the synergy of the multiple pharmacological actions of SBs. Regardless of the predictions of this information, complementary interactions obtained by the main compound acting on multiple targets and pathways and the synergistic effects on multiple pathologies based on this should be the focus of research, and experimental and computational biological studies that consider this aspect will be essential in the future. The value of this study is that we revealed that SB is a potential candidate for the treatment of OA, warranting a detailed follow-up, as described above.

## 4. Materials and Methods

The study consisted of high-performance liquid chromatography (HPLC), a monosodium iodoacetate (MIA)-induced OA model, an acetic acid-induced writhing response model, and lipopolysaccharide (LPS)-stimulated RAW264.7. Based on the results of the network pharmacology analysis to identify the active ingredients that are likely to be effective against arthritis, the SB extract was analyzed by HPLC to quantify the amount of each active ingredient in the samples used in this study. The MIA-induced arthritis model causes cartilage damage by inhibiting the corresponding action of chondrocytes when MIA, a substance that reduces cellular glyceraldehyde-3-phosphate dehydrogenase activity, is injected into the joint cavity. This model is very similar to the findings in human OA and is widely used to evaluate drugs for OA because of the advantage that chondrocyte damage occurs in proportion to the concentration of the MIA injection, making it easy to control the timing and extent of the lesion. The acetic acid-induced writhing response model is a model in which acetic acid is injected into the peritoneal cavity to induce endogenous pain, resulting in a writhing response, and it was evaluated as the most appropriate model to quantify pain and determine the extent to which it improves pain, which is very important in arthritis. The cell model, in which RAW264.7 was treated with LPS to induce inflammation, was designed to check the effectiveness of improving inflammation, another pathological process in arthritis, and the expression level of inflammatory factors.

### 4.1. Network Pharmacology of SB for OA

#### 4.1.1. Active Compounds of SB- and OA-Related Target Genes

Potential active compounds and target proteins of SB were searched for in four databases: the traditional Chinese medicine systems pharmacology database and analysis platform (https://old.tcmsp-e.com/tcmsp.php), traditional Chinese medicine information database (http://bidd.group/TCMID/), encyclopedia of traditional Chinese medicine (http://www.tcmip.cn/ETCM/), and high-throughput experiment- and reference-guided database of traditional Chinese medicine (http://herb.ac.cn/) [54,55,56,57]. After removing duplicates of the compounds and target proteins collected from these databases, the gene names of the target proteins (“*Homo Sapiens*” as the retrieval species) were standardized using the UniProt database (https://www.uniprot.org/) [58]. Data on OA-related target genes were retrieved from the DrugBank (https://go.drugbank.com/) and GeneCards databases (http://www.genecards.org), with “osteoarthritis” as the keyword. For targets in GeneCards, only those with a score ≥ 1 were screened [59].

#### 4.1.2. Common Target Acquisition

Venn diagrams of common targets of SB and OA were created using Bioinformatics and Evolutionary Genomics (https://bioinformatics.psb.ugent.be/webtools/Venn). Elements of the SB and OA targets were imported into Cytoscape (version 3.9.1; https://cytoscape.org/) to graphically represent the drug compound–target–disease network (D-C-T-D network). The degree of each node was calculated using a layout tool; the greater the degree, the larger the number of nodes in the network.

#### 4.1.3. Protein–Protein Interaction (PPI) Network Construction

For the identified common targets, a PPI network was generated using the String database (version 11.5; https://string-db.org/), and the “minimum required score with the highest confidence (0.900)” was defined for the PPI. For the topological study of the PPI network, the PPI network was acquired, unrelated protein nodes were removed, and the data were loaded into Cytoscape 3.9.1. Genes with above-average degree centralities were selected as hub targets.

#### 4.1.4. Gene Ontology (GO) and Kyoto Encyclopedia of Genes and Genomes (KEGG) Analysis

Metascape (https://metascape.org/gp/index.html) incorporates over 40 gene functional annotation databases into a web-based tool for gene enrichment analysis [60]. GO and KEGG analyses were performed using the Metascape tool. By specifying the species as “*Homo sapiens*”, setting the cut-off *p*-value at 0.01, requiring a minimum overlap of three for enrichment analysis, and encompassing biological processes, cellular components, molecular functions, and KEGG pathways, we investigated the gene symbols of common targets in Metascape.

### 4.2. Scutellaria baicalensis Georgi Extract Preparations

Dried SB Georgi roots were obtained from Yaksudang Pharmaceutical Co., Ltd. (Seoul, Republic of Korea). They were authenticated by Donghun Lee, and voucher specimens were deposited in the Department of Herbal Pharmacology, College of Korean Medicine, Gachon University (No. 2009150001). Each SB root was powdered to 10 g at an equal weight ratio, then extracted using 30% EtOH at 100 °C for 3 h under reflux with 10 times the amount of water. The filtered extract was concentrated under decreased pressure and then lyophilized at −80 °C. The yield was 43.4%.

### 4.3. High-Performance Liquid Chromatography 

High-performance liquid chromatography was performed using the Agilent 1100 HPLC system (Agilent Technologies, Santa Clara, CA, USA). A Luna C_18_ column (5 µm particle size, 250 mm × 4.6 mm; Phenomenex, Torrance, CA, USA) maintained at 30 °C was used to perform chromatic separation. The mobile phase consisted of 0.1% formic acid (A) and acetonitrile (B) with a flow rate of 1.0 mL/min. The gradient elution of solvent B was as follows: 0 to 10 min, 20% to 30%; 10 to 20 min, 30% to 33%; 20 to 30 min, 33% to 60%; 30 to 35 min, 60% to 100%. The injection volume was 10 µL, and detection occurred at 275 nm. The extract was analyzed in triplicate.

### 4.4. Animal

Male Sprague–Dawley rats (five weeks old, 200 ± 10 g) and ICR mice (six weeks old, 35 ± 5 g) were purchased from DBL (DBL, Incheon city, Republic of Korea). The animals used in all experiments were 45 rats for the OA-induced model and 40 mice for the writhing and hot plate test. The animals were housed under the following animal room conditions: 20–24 °C, 45–65% humidity, and a 12 h light/dark cycle for at least 7 days. The animals had free access to food and water. Ethical regulations for animal handling and care at Gachon University (GIACUC-R2020028) were rigorously adhered to during all animal experimental processes.

### 4.5. Monosodium Iodoacetate Injection and Diet Preparation

This experiment was conducted to establish a rat model for MIA induction [14,61,62] (Table 4). There were five groups with nine rats in each group. The groups consisted of sham, control, indomethacin, SB at low doses, and SB at high doses. Control, indomethacin, SB low dose, and SB high dose were administered 50 μL intra-articular injections of 40 mg/mL MIA (Sigma-Aldrich Inc., St. Louis, MO, USA) to induce OA after being sedated with a 2% isofluorane and O_2_ mixture. In the Sham group, saline was injected 50 μL into the knee joint cavity. The sham and control groups were administered DW daily for 24 days. P.O. indomethacin (Sigma, USA) was dissolved in distilled water (DW) at a dose of 3 mg/kg and administered daily for 24 days. Low-dose (80 mg/kg) and high-dose (240 mg/kg) SB were dissolved in DW and administered orally daily for 24 days. All groups administered samples P.O. and dissolved in DW at a dose of 10 mL/kg using an oral zonde needle (JD-S-124-50-18G; Jeong do bio&plant Co., Ltd., Seoul, Republic of Korea). At the end of the experiment, the rats were euthanized with CO_2_.

### 4.6. Hind Limb Weight-Bearing Measurement

The weight-bearing capacity of the hind limb was measured using an incapacitance meter tester 600 (IITC Life Science Inc., Woodland Hills, CA, USA) on days 0, 3, 7, 10, 14, 17, 21, and 24 h after OA induction in Sprague–Dawley rats. The weight-bearing capacity of the MIA-induced rats was calculated by weighing each hind limb after each group of rats was administered daily and placed on an incapacitance meter for 10 s for 24 days to calculate the improvement in the weight-bearing capacity of each sample. The weight-bearing ratio of the right hind limb was estimated using the following formula:

$$weight\;bearing\;ratio\left(\%\right)=\frac{weight\;of\;right\;hind\;limb}{weight\;of\;right\;and\;left\;hind\;limbs}\times 100$$

### 4.7. Cartilage Degradation Evaluation

Following the sacrifice of the rat, the right knees were disarticulated and scanned for macroscopic scoring. The knee of the MIA-induced model was photographed using a digital camera (α6600; Sony Group Corp., Tokyo, Japan). The articular cartilage erosion was assessed using the macroscopic scoring technique [63,64] (Table 5).

### 4.8. Monosodium Iodoacetateserum Concentration Analysis

An abdominal vein was used to draw whole blood, which was then left to clot for 30 min. The whole blood was centrifuged for 10 min at 4000 rpm, and the isolated serum was kept at −80 °C for storage. A quantitative analysis of serum IL-1β, TNF-α, and IL-6 concentrations was performed in duplicate using a sandwich assay in a 96-well plate, based on the manufacturer’s instructions (R&D Systems, Minneapolis, MN, USA, RND-LXSARM-03). 

### 4.9. Writhing Test

There were four groups with eight male ICR mice in each group. The groups consisted of control, ibuprofen, SB at low doses, and SB at high doses. The control group was fed water, and the positive control group was orally fed 200 mg/kg ibuprofen (Sigma, USA) and SB (160 and 480 mg/kg). Ibuprofen was used as a positive control. Thirty minutes after oral treatment, 0.7% acetic acid (10 mL/kg; Sigma, USA) was intraperitoneally injected, and writhing responses were measured 10 min later. Following the enlargement of the hind limbs, the twisting reaction consisted of turning the pelvis and contracting the abdominal wall. Experiments were repeated twice. All groups administered samples P.O. and dissolved in DW at a dose of 10 mL/kg using an oral zonde needle (JD-S-124-50-22G; Jeong do bio&plant Co., Ltd., Seoul, Republic of Korea). At the end of the experiment, the mice were euthanized with CO_2._

### 4.10. Hot Plate Test

There were four groups with eight male ICR mice in each group. The groups consisted of control, morphine, SB at low doses, and SB at high doses. The control group was fed water, and the positive control group was intraperitoneally injected with 10 mg/kg morphine. SB was orally fed SB 160 and 480 mg/kg. Morphine (Myungmoon Pharm Co., Ltd., Seoul, Republic of Korea) was used as a positive control. The sample was administered 30 min before, and every 30 min, the mice were placed on the hot plate for 30 s to check for licking, jumping, and shaking responses. The plate temperature was 55 °C. We recorded the time of the first response. All groups administered samples P.O. and dissolved in DW at a dose of 10 mL/kg using an oral zonde needle (JD-S-124-50-22G; Jeong do bio&plant Co., Ltd., Seoul, Republic of Korea). At the end of the experiment, the mice were euthanized with CO_2_.

### 4.11. RAW264.7 Cell Culture

The Korean Cell Line Bank (Seoul, Republic of Korea) obtained RAW264.7 cells. Complete medium including DMEM with 10% FBS, 100 IU/mL penicillin, and 100 µg/mL streptomycin was used, and the cells were kept at 37 °C and 5% CO_2_ (Gibco BRL, Grand Island, NY, USA).

### 4.12. Nitric Oxide (NO) Production and Cell Toxicity Evaluation

The RAW264.7 cells, seeded at 2 × 10^4^/well, were incubated at 37 °C and 5% CO_2_ for 24 h. Following seeding, the cells were treated for 24 h with varying doses of SB (10–300 µg/mL) and lipopolysaccharide (LPS; 500 ng/mL). Dexamethasone (Sigma, USA) was used as a positive control. The culture supernatant was mixed with Griess reagent (1:1; Sigma, USA), and the absorbance at 540 nm was measured. The MTT assay was used to evaluate cytotoxicity. RAW264.7 cells were treated with 5 mg/mL MTT reagent for 1 h at 37 °C with 5% CO_2_. After the supernatant was removed, 100 µL of DMSO was added and left for 10 min before determining the absorbance at 540 nm. All experiments were repeated thrice.

### 4.13. Quantitative Real-Time Polymerase Chain Reaction (qRT-PCR) Analysis

Total RNA was extracted from OA-induced cartilage tissue and LPS-stimulated RAW264.7 cells using an AccuPrep^®^ Universal RNA Extraction Kit (Bioneer, Daejeon, Republic of Korea) and reverse transcribed into cDNA using CycleScript™ RT Pre&Master Mix (Bioneer, Republic of Korea), according to the manufacturer’s protocol. mRNA expression was quantified with 2X-GreenStar™ qPCR MasterMix (Bioneer, Republic of Korea). All experiments were repeated thrice. The relative gene expression was determined using the threshold cycle (CT) method, and the fold changes were calculated using the 2^−ΔΔCT^ formula. The primer sequences consisted of exons and are listed in the following Table 6 and Table 7:

### 4.14. Protein Expression Analysis

For the protein analysis, cartilage from the right knee joint where MIA was injected was harvested after sacrificing the MIA rats and stored at −80 °C. The protein expression of matrix metalloproteinases (MMPs) (MMP-1, 3, 8, and 13), IL-1β, NF-κB p65, NOS2, and β-actin was performed using a Western blot assay (Table 8). The total protein from the OA-induced cartilage and LPS-stimulated RAW246.7 cells was extracted using the cOmplete™ EDTA-free Protease Inhibitor Cocktail (Sigma, USA) and RIPA buffer (Cell Signaling Technology Inc., Danvers, MA, USA) with a homogenizer (Benchmark, D1000-E; Sayreville, NJ, USA). Equal amounts of samples were subjected to sodium dodecyl sulfate-polyacrylamide gel electrophoresis, and the separated proteins were transferred to polyvinylidene difluoride membranes with a Semidry Transfer Cell (Bio-Rad Laboratories, Inc., Hercules, CA, USA) for 1 h at 15 V. To prevent nonspecific antibody binding, the membranes were incubated with blocking buffer (Bio-Rad, USA) for 30 min at room temperature. The membrane was then washed thrice with TBST (BioRad, USA) before being exposed to the primary antibodies (MMPs, IL-1β, NF-κB, NOS2, and β-actin) for 24 h at 4 °C. The antibodies were obtained from Abcam and Cell Signaling Technology (Danvers, MA, USA). A secondary antibody was used to probe the membrane at room temperature for 1 h, and then Clarity^TM^ Western ECL Substrate solution (Bio-Rad Laboratories, Inc.) was used for the reaction. Western blotting was performed using Azure 280 (Azure Biosystems, Dublin, CA, USA). Experiments were repeated thrice.

### 4.15. Statistics

GraphPad Prism^®^ 9.0 (GraphPad Software, San Diego, CA, USA) was used for the statistical analysis, which included a one-way analysis of variance and Dunnett’s post hoc test. Measurements were reported as the mean standard error of the mean, and significance was indicated at *p* < 0.05.

## 5. Conclusions

The major compounds (wogonin, baicalein, and acacetin) and hub targets (JUN, RELA, FOS, TP53, and MAPK14) of SB in OA were explored based on network pharmacology predictions. According to the KEGG and GO enrichment studies, the PI3K-Akt signaling pathway, fluid shear stress, atherosclerosis pathway, and cellular senescence pathway play critical roles in the relief of OA caused by SB. The ability of SB to reduce pain, inflammation, and joint damage in OA joints has been demonstrated both in vivo and in vitro, supporting the findings of a network pharmacology study. To the best of our knowledge, this study is the first attempt from an integrated perspective to decipher the mechanism of action of SB in the treatment of OA through multiple compounds, targets, and pathways, thereby clinically applying SB to OA management.

## Figures and Tables

**Table 1:** Chemical compounds of *Scutellaria baicalensis* extracts.

Pubchem ID	Compound Name	Structure	OB (%)	DL
6782	Diisobutyl phthalate	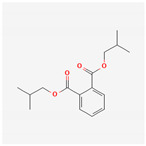	43.59	0.35
31161	Pedalitin	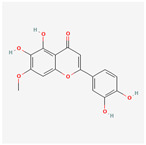	34.02	0.31
33934	Diisooctyl phthalate	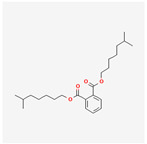	43.59	0.39
64982	Baicalin	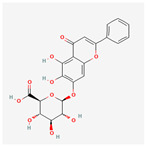	40.12	0.75
72322	Coptisine	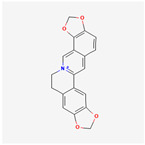	30.67	0.86
72323	Jatrorrhizine	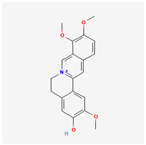	30.44	0.75
124211	Skullcapflavone II	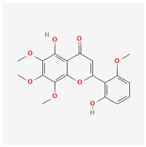	69.51	0.44
156992	5,8,2′-Trihydroxy-7-methoxyflavone	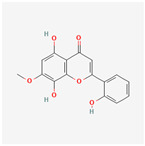	37.01	0.27
159029	Tenaxin I	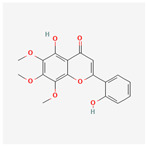	31.71	0.35
160876	Epiberberine	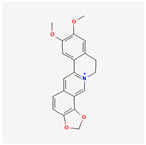	43.09	0.78
161271	Salvigenin	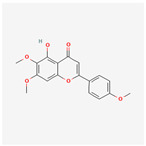	49.07	0.33
182232	(+)-Epicatechin	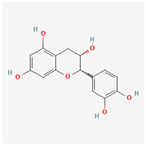	48.96	0.24
188308	Carthamidin	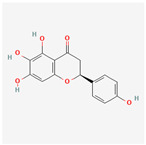	33.23	0.24
188316	5-Hydroxy-7,8-dimethoxyflavone	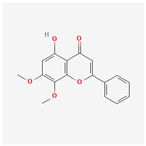	44.09	0.25
222284	beta-Sitosterol	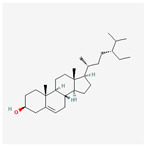	36.91	0.75
373261	Eriodyctiol (flavanone)	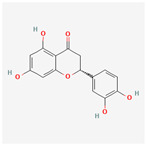	41.35	0.24
440735	Eriodictyol	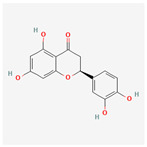	71.79	0.24
457801	Clionasterol	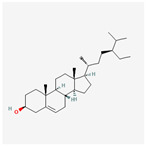	36.91	0.75
471719	Negletein	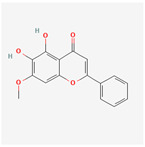	41.16	0.23
5280442	Acacetin	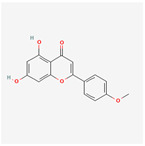	34.97	0.24
5280666	Chrysoeriol	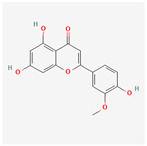	35.85	0.27
5280794	Stigmasterol	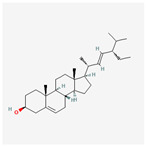	43.83	0.76
5281330	Poriferasterol	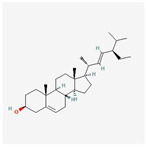	43.83	0.76
5281605	Baicalein	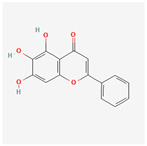	33.52	0.21
5281674	Norwogonin	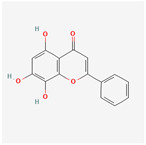	39.4	0.21
5281703	Wogonin	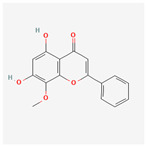	30.68	0.23
5283637	22,23-Dihydrobrassicasterol	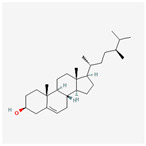	37.58	0.71
5320315	Oroxylin A	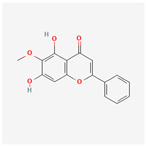	41.37	0.23
5320399	Skullcapflavone I	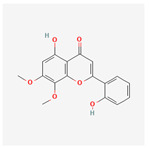	76.26	0.29
5321865	5,7,2′,6′-Tetrahydroxyflavone	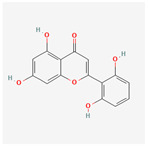	37.01	0.24
5322059	Viscidulin II	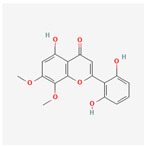	45.05	0.33
42608119	5,7,4′-trihydroxy-8-methoxyflavanone	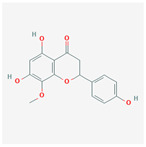	74.24	0.26
5322078	4′-Hydroxywogonin	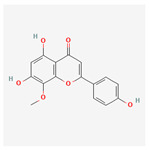	36.56	0.27
5365674	11,13-Eicosadienoic acid, methyl ester	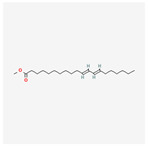	39.28	0.23
5367328	1-Monolinolenoyl-rac-glycerol	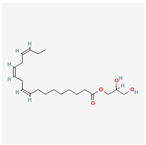	38.14	0.31
9601691	Glucobrassicin	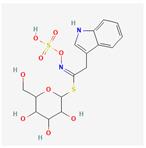	66.02	0.48
12303645	3-epi-beta-Sitosterol	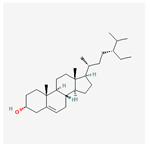	36.91	0.75
13889022	Rivularin (flavone)	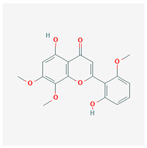	37.94	0.37
14135323	(2S)-dihydrobaicalein	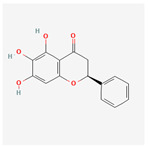	40.04	0.21
25721350	Dihydrooroxylin	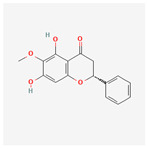	66.06	0.23
26213330	(2R)-5,7-dihydroxy-2-(4-hydroxyphenyl)-6-methoxy-2,3-dihydrochromen-4-one	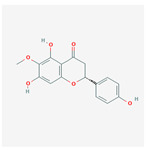	36.63	0.27
44258628	5,7,3′,6′-Tetrahydroxy-6,8,2′-trimethoxyflavone	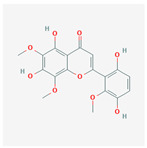	33.82	0.45
141457867	5-Hydroxy-2-(2-hydroxy-5-methoxyphenyl)-6,7,8-trimethoxychromen-4-one	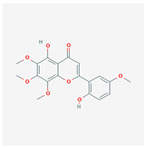	104.34	0.44
162988960	Carthamidin	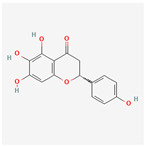	41.15	0.24

DL: drug-likeness, OB: Oral bioavailability.

**Table 2:** Top 15 compounds of SB from the Network Analyzer.

Compound Name	Degree Centrality	Betweenness Centrality	Closeness Centrality
Wogonin	26	0.094	0.509
Baicalein	19	0.062	0.471
Acacetin	16	0.035	0.456
beta-Sitosterol	13	0.030	0.442
5-Hydroxy-7,8-dimethoxyflavone	12	0.011	0.438
Oroxylin A	12	0.017	0.438
4′-Hydroxywogonin	11	0.009	0.433
Chrysoeriol	11	0.009	0.433
5-Hydroxy-2-(2-hydroxy-5-methoxyphenyl)-6,7,8-trimethoxychromen-4-one	10	0.010	0.429
Rivularin (flavone)	9	0.007	0.425
Jatrorrhizine	8	0.004	0.421
Salvigenin	8	0.004	0.421
Skullcapflavone I	8	0.003	0.421
Tenaxin I	8	0.003	0.421
Viscidulin II	8	0.003	0.421

**Table 3:** The topological characteristics of hub targets from SB against OA.

Gene Name	Degree Centrality	Betweenness Centrality	Closeness Centrality	Average Shortest Path Length
*JUN*	22	0.209	0.560	1.786
*RELA*	16	0.086	0.519	1.929
*FOS*	15	0.060	0.494	2.024
*TP53*	15	0.222	0.525	1.905
*MAPK14*	13	0.058	0.500	2.000
*ESR1*	13	0.111	0.472	2.119
*TNF*	12	0.078	0.483	2.071
*IL6*	11	0.112	0.477	2.095
*AKT1*	10	0.067	0.457	2.190
*NR3C1*	9	0.008	0.457	2.190
*AR*	8	0.015	0.412	2.429
*CCND1*	7	0.004	0.438	2.286
*CXCL8*	7	0.027	0.457	2.190
*TGFB1*	7	0.033	0.429	2.333

**Table 4:** Monosodium iodoacetate-induced OA model design.

Group	OA Inducer (50 μL, Intra-Articular)	Sample (10 mL/kg, P.O.)
Sham	Saline	DW
Control	MIA 40 mg/mL	DW
Indomethacin	MIA 40 mg/mL	indomethacin 200 mg/kg
SB (low dose)	MIA 40 mg/mL	SB 80 mg/kg
SB (high dose)	MIA 40 mg/mL	SB 240 mg/kg

**Table 5:** Macroscopic score of cartilage erosion.

Grade	Cartilage Appearance
0	Normal appearance in cartilage surface
1	Slight yellowish discoloration of the surface or slight fibrillation
2	Erosion reaching the superficial or middle layers of the cartilage
3	Extensive erosions reaching down to the subchondral bone
4	Massive erosions with extensive exposure of subchondral bone

**Table 6:** mRNA primer sequence for OA-induced cartilage tissues.

MMP-1	F	AACTTGGGTGAAGACGTCCA
	R	TCCTGTCACTTTCAGCCCAA
MMP-3	F	GTACGGCTGTGTGCTCATCC
	R	TCAGCCCAAGGAACTTCTGC
MMP-8	F	TCTGTTCTTCTTCCACACACAG
	R	GCAATCATAGTGGCATTCCT
MMP-13	F	ACCTTCTTCTTGTTGAGTTGGA
	R	CTGCATTTCTCGGAGTCTA
JUN	F	CCAACCAACGTGAGTGCAAG
	R	GAG GGCATCGTCGTAGAAGG
FOS	F	TACTACCATTCCCCAGCCGA
	R	GCGTATCTGTCAGCTCCCTC
IL-1β	F	AACTCAACTGTGAAATAGCAGC
	R	TCCACAGCCACAATGAGTG
TNF-α	F	GCATGATCCGAGATGTGGAA
	R	GATGAGAGGGAGCCCATTTG
IL-6	F	TCCGCAAGAGACTTCCAGC
	R	CCTCCGACTTGTGAAGTGG
COX-2	F	GTTCCAACCCATGTCAAAAC
	R	TGTCAGGAATCTCGGCGTAG
Ptger2	F	TGTGTGTACTGTCCGTCTGC
	R	CAGGGATCCAGTCTCGGTGT
TGF-β1	F	AGGAGACGGAATACAGGGCT
	R	CCACGTAGTAGACGATGGGC
Type II collagen	F	TGGCCTTGGTGGAGGAAA
	R	AGGACCAGGGAGGCCTCTTT
IL-4	F	CGTGATGTACCTCCGTGCTT
	R	GTGAGTTCAGACCGCTGACA
TIMP-1	F	TTTCCCTGTTCAGCCATCCC
	R	TAGCCCTTCTCAGAGCCCAT
GAPDH	F	CTTGTGACAAAGTGGACATTGTT
	R	TGACCAGCTTCCCATTCTC

The mRNA primer sequence used the *Rattus norvegicus* gene. MMP: matrix metalloproteinase, JUN/FOS: gene of activator protein-1, IL: interleukin, TNF: tumor necrosis factor, COX: cyclooxygenase, Ptger2: prostaglandin E receptor 2, TGF: transforming growth factor, TIMP: tissue inhibitor of metalloproteinase, GAPDH: Glyceraldehyde 3-phosphate dehydrogenase.

**Table 7:** mRNA primer sequence for LPS-stimulated RAW264.7 cells.

MMP-1	F	ATGCCTAGCCTTCCTTTGCT
	R	TTCCAGGTATTTCCAGACTG
MMP-3	F	AAGTTCCTCGGGTTGGAGAT
	R	ACCAACATCAGGAACACCAC
MMP-8	F	CAATCAATTCCGGTCTTCGA
	R	GGTTAGCAAGAAATCACCAGA
MMP-13	F	AACCAAGATGTGGAGTGCCT
	R	GACCAGACCTTGAAGGCTTT
JUN	F	ACAGAGCATGACCTTGAACCT
	R	GTGATGTGCCCATTGCTGGA
FOS	F	GGACTTTTGCGCAGATCTGT
	R	GGTGGGGAGTCCGTAAGGAT
IL-1β	F	CCAGCTTCAAATCTCGCAGC
	R	GTGCTCATGTCCTCATCCTGG
TNF-α	F	GAGAAGTTCCCAAATGGCCT
	R	AGCCACTCCAGCTGCTCCT
IL-6	F	CACTTCACAAGTCGGAGGCT
	R	CAAGTGCATCATCGTTGTTC
COX-2	F	ATCCATGTCAAAACCGTGGG
	R	TTGGGGTGGGCTTCAGCAG
Ptger2	F	CTGGTAACGGAATTGGTGC
	R	TGGCCAGACTAAAGAAGGTC
NOS2	F	ACCAAGATGGCCTGGAGGAA
	R	CCGACCTGATGTTGCCATTG
TGF-β1	F	GGACTCTCCACCTGCAAGAC
	R	TGTTGTACAAAGCGAGCACC
IL-4	F	ACGGAGATGGATGTGCCAA
	R	TGCGAAGCACCTTGGAAGC
GAPDH	F	ATGGTGAAGGTCGGTGTG
	R	GCCGTGAGTGGAGTCATAC

The mRNA primer sequence used the *Mus musculus* gene. MMP: matrix metalloproteinase, JUN/FOS: gene of activator protein-1, IL: interleukin, TNF: tumor necrosis factor, COX: cyclooxygenase, Ptger2: prostaglandin E receptor 2, TGF: transforming growth factor, TIMP: tissue inhibitor of metalloproteinase, GAPDH: Glyceraldehyde 3-phosphate dehydrogenase.

**Table 8:** Antibodies.

Antibody	Dilution Rate	Company
MMP-1	1:700	Proteintech
MMP-3	1:1000	Abcam
MMP-8	1:1000	Abcam
MMP-13	1:2000	Proteintech
IL-1β	1:1000	Abcam
NF-κB p65	1:1000	Cell Signaling
NOS2	1:1000	Abcam
β-actin	1:1000	Cell Signaling

## Data Availability

The original contributions presented in this study are included in the article/ further inquiries can be directed to the corresponding author.
